# Research on Risk Evaluation of Internet Strategic Transformation of Manufacturing Enterprises Based on the BP Artificial Neural Network

**DOI:** 10.3389/fpsyg.2022.945957

**Published:** 2022-07-22

**Authors:** Huang Honglei, Ghulam Hussain Khan Zaigham

**Affiliations:** ^1^School of Economics and Management, Hubei Engineering University, Xiaogan, China; ^2^Department of Management, COMSATS University, Islamabad, Pakistan

**Keywords:** BP artificial neural network, manufacturing enterprise, Internet strategic transformation, risk evaluation, risk

## Abstract

For manufacturing enterprises to successfully enter the “Industry 4.0” era and establish advantages in the new wave of the Industrial Revolution, they must use Internet thinking to transform manufacturing enterprises and promote the in-depth integration of informatization and industrialization under the premise of managing and controlling risks, to achieve transformation and upgrading. Research on and management of the risks of manufacturing enterprises’ Internet strategic transformation directly affects the success or failure of enterprises’ transformation. This study constructed a risk evaluation model for a manufacturing enterprise’s Internet strategic transformation based on the back propagation (BP) artificial neural network and conducted a case study on one enterprise.

## Introduction

Scholars have conducted extensive research on the risks of corporate strategic transformation. In terms of the types of strategic transformation risk, identification of risk factors, mechanism of action, and sources, Baird and Thomas (1985) (p.10) divided strategic transformation risks into macro-environmental risks, decision-makers’ risks, industry risks, strategic issues risks, and organizational risks and analyzed them in detail. Sayn and Don (1999) (p.24) conducted research on the mechanism of strategic transformation risk and the identification model of risk factors. Scholars such as Winfrey and Budd (1997) (p.62) analyzed the three main sources of strategic risk by constructing a multi-dimensional model of strategic risk: innovation risk, operational risk, and competitive risk. Slywoztky (2007) (p.10) conducted an in-depth discussion on the composition of strategic transformation risks for the first time in a 2004 study and summed up the reasons leading to strategic transformation risks. Markowitz (1987) proposed the variance-standard deviation method to measure risk, which has strong applicability, greater influence, and wider application.

Domestic scholars have conducted research on corporate strategic transformation risks from different perspectives, such as the source, formation mechanism, identification, and evaluation. In terms of research on the sources of corporate strategic transformation risks, Yu (2003) (p.2) analyzed the direct causes of transformation risks: the ability to organize, control, and identify core capabilities. Zhang (2013) (p.3) started with the internal connection between resources and strategy and proposed that the strategic transformation risks of small- and medium-sized manufacturing enterprises mainly come from the uncertain factors in the process of resource changes that match the corporate strategic transformation: resource matching risks, resource inertia risk, and organizational flexibility risk.

Regarding research on the formation mechanism of corporate strategic transformation risk, Sun and Cheng (2012) (p.8) made relevant suggestions from the perspective of capability. In terms of research on the identification and evaluation of corporate strategic transformation risks, Xiang et al. (2012) (p.3) discussed the identification and evaluation of risks they faced during strategic transformation in the form of cases and put forward relevant recommendations. Liu (2015) (p.1) addressed the problem of identifying risk factors for corporate strategic transformation in risk-related situations and proposed a DEMATEL-based method for identifying risk factors. With this method, core risk factors and types of risk factors can be identified, and each risk is calculated. The comprehensive evaluation value of factors. Scholars such as Su and Fuzzy (2012) (p.2) focused on small- and medium-sized enterprises as a research object, identified the risks arising from their strategic transformation, constructed their risk index system based on this, and further adopted a comprehensive evaluation method for risks. Scholars such as Wu and Xiang (2012) (p.8) focused on innovative companies as a research object and constructed their strategic transformation risk indicator system. Xu et al. (2014) (p.1) argued that manufacturing companies will be bound by different types of risks during the transition to services. Based on the identification of risk factors, methods such as the entropy weight method and fuzzy mathematics are used to evaluate risks.

In terms of corporate Internet strategic transformation, Yu and Gao (2018) (p.10) used the embedded multi-case analysis method to explore the ecological transformation path of Chinese manufacturing enterprises in the context of the industrial Internet. Liu (2020) (p.11) argued that there are ideological differences between the Internet industry and traditional industries, which makes traditional enterprises’ Internet transformation difficult; based on the development status of traditional enterprises, he proposed an Internet transformation route for traditional enterprise business models, management models, and marketing models.

In summary, the existing research on the risks of corporate strategic transformation and corporate Internet transformation is relatively rich, while studies on the risks of manufacturing corporate strategic transformation are relatively few. Moreover, there is clearly insufficient research on the risks of manufacturing corporate Internet strategic transformation.

## Construction of a Risk Evaluation Index System for Manufacturing Enterprises’ Internet Strategic Transformation

This study aimed to construct a risk evaluation index system based on the causes and types of risks in manufacturing enterprises’ Internet strategic transformation. Three perspectives were considered:: flexible manufacturing, technological innovation, and management development, This system comprises the following layers:

The target layer refers to the overall goal to be achieved in the risk evaluation of the manufacturing enterprise’s Internet strategic transformation, that is, to accurately measure the risk level of the manufacturing enterprise’s Internet strategic transformation, dynamically reflect the development and change trend of risks in the manufacturing enterprise’s Internet strategic transformation, and improve the accuracy and effectiveness of decision implementation.

The criterion layer divides the goals into multiple basic categories according to the type of work content and the nature and process of risk evolution of manufacturing enterprises’ Internet strategic transformation. This article mainly includes the following three types of risks: flexible manufacturing risks, technological innovation risks, and management development risks.

The element layer is a further refinement of the criterion layer and is the subdivision dimension of the measure criterion layer. This article mainly includes six subdivision dimensions: equipment flexibility, organization flexibility, intelligent technology, Internet technology, flexible management, and financing management.

The indicator layer refers to the set of basic evaluation indicators for the risk assessment of the Internet strategic transformation of manufacturing enterprises, including multiple indicators, such as high main equipment costs, high equipment sinking costs, and strong dependence on relationships.

**FIGURE 1:**
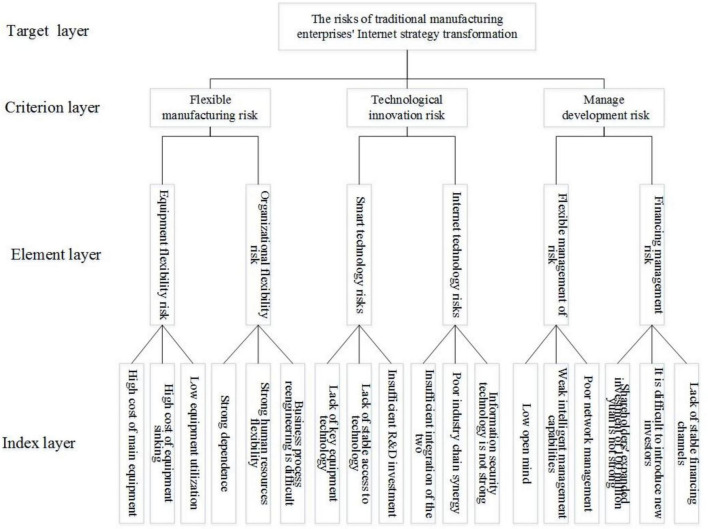
Structural diagram of the risk evaluation index system for the Internet strategic transformation of manufacturing enterprises.

Figure 1 shows the structure of the risk evaluation index system for manufacturing enterprises’ Internet strategic transformation.

### Flexible Manufacturing Risks

#### Equipment Flexibility Risk

Equipment flexibility risk refers to the uncertainty arising from whether the original equipment, newly purchased equipment, and other equipment resources of the manufacturing enterprise can well meet the needs of the new strategic development in the process of Internet strategic transformation, including the high cost of the company’s main equipment and equipment. Three three-level indicators such as high sunk cost and low equipment utilization. The high cost of the main equipment means that the main equipment used by the company is of greater value, accounting for 50% or more of the company’s assets. The high cost of equipment sinking means that the original equipment of the enterprise cannot be effectively used after the transformation, and the equipment loss reaches 50% or more. The low utilization rate of equipment refers to the poor compatibility between new equipment purchased by enterprises in transformation and old equipment, and the production capacity of new equipment is at or below 60%.

#### Organizational Flexibility Risk

Organizational flexibility risk refers to the uncertainty caused by whether a manufacturing enterprise can successfully redesign business processes and overcome resistance such as dependencies to meet various transformation needs of the enterprise during the process of Internet strategic transformation, including strong relationships dependence, weak human resource flexibility, business process re-engineering difficulties, and other three-level indicators. Strong relationship dependence means that the company’s original major customers and government and other relationship resources cannot be fully utilized after transformation, and the utilization rate is 60% or less. Weak human resource flexibility refers to the lack of change in the importance of human emotion, personality, desire, ability, and other factors required to adapt to the needs of Internet transformation. Difficulty in business process re-engineering refers to the fact that the business process of an enterprise is highly professional, and the cost of its re-engineering accounts for 30% or more of the value of enterprise assets.

### Technological Innovation Risk

#### Smart Technology Risk

Smart technology risk refers to the uncertainty caused by whether a manufacturing company can master smart technology to meet the needs of corporate transformation during the process of Internet strategic transformation, including three three-level indicators: lack of key equipment technology, lack of stable access to technology, and insufficient R&D investment. Lack of key equipment technology means that the key equipment technology required for enterprise transformation is not yet available or not fully mastered. Lack of stable access to technology means that the company has neither a dedicated R&D department, nor a fixed cooperative R&D institution, and the access to technology is less and unstable. Insufficient R&D investment means that the company’s investment in technology R&D accounts for less than 6% of sales revenue.

#### Internet Technology Risk

Internet technology risk refers to the uncertainty in the use of Internet technology in the transformation of enterprise production models, business models, and marketing models due to insufficient Internet technology during the process of Internet strategic transformation, including three three-level indicators: insufficient integration of industrialization, poor coordination of the industrial chain, and weak information security technology. Insufficient integration of industrialization and industrialization means that the integration of industrialization and industrialization has not achieved relevant results in terms of technology integration, product integration, business integration, and industrial derivation. Poor coordination of the industrial chain means that the number of enterprises in the upstream and downstream enterprises of the industrial chain where the enterprise is located is less than 50% of the enterprises carrying out the strategic transformation of the Internet. Weak information security technology means that the enterprise does not have the network security technology and corresponding talents required for the strategic transformation of the Internet.

### Management Development Risk

#### Flexible Management Risk

Flexible management risk refers to the uncertainty caused by whether the flexible management capabilities can meet these transformation needs during the transformation of business models, marketing models, and especially management models, including three three-level indicators: low mental openness, weak intelligent management ability, and poor network management ability. Low mental openness means that the proportion of employees who have broad vision, open thinking, and highly approve of the corporate Internet strategy transformation is less than 50%. Weak intelligent management ability means that the company does not have the conditions and capabilities to realize intelligent management of production, marketing, logistics, etc. Poor network management ability means that companies do not have the network management capabilities required for the strategic transformation of the Internet.

#### Financing Management Risk

Financing management risk refers to the uncertainty caused by whether manufacturing companies can raise funds for transformation in a timely manner during the process of Internet strategic transformation, including a lack of willingness of shareholders to expand investment, the difficulty of introducing new investors, and the lack of stable financing channels. The lack of willingness of shareholders to expand investment means that the proportion of shareholders who are willing to make additional investment for the company’s Internet strategic transformation is less than 50%. The difficulty of introducing new investors means that companies have not yet found new investors who are interested in investing in the corporate Internet strategy transformation. The lack of stable financing channels means that companies do not have relatively fixed financing partners and lack stable financing channels.

## Construction of Risk Evaluation Model for Manufacturing Enterprises’ Internet Strategic Transformation

### Back Propagation Artificial Neural Network Evaluation Model

**FIGURE 2:**
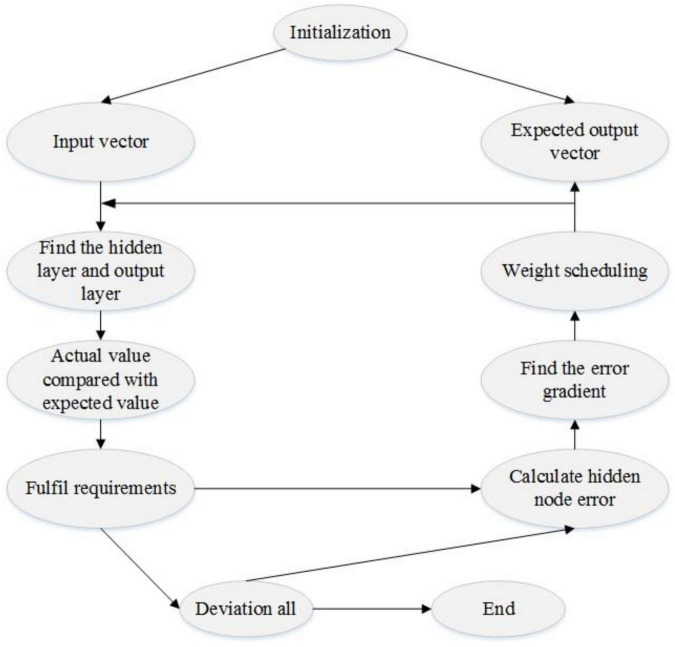
BP artificial neural network risk assessment flowchart.

The structure of a back propagation (BP) artificial neural network mainly comprises an input layer, output layer, and hidden layer. Among them, the hidden layer can be one or multiple layers. In the forward propagation neural network BP, the signal input into the input layer neuron hidden layer, until the signal processing, directly into the output layer, and the layer neural element affects only the current state of the next layer, and it will not affect the state of neurons in other layers. If the output state of the output layer does not reach a desired value, the error signal terminates forward propagation, propagation state into reverse, starting from the start-stop state, the connection weights for the weight of each layer is adjusted to meet the requirements up to the desired value will stop. The algorithm uses a large amount of training on samples, along the direction of the negative gradient of the error function, and uses the fastest descent method to adjust the weight value, so that the error value converges to the minimum value, which is the process of finding the minimum value of the error function, as shown in Figure 2.

### Risk Evaluation Model of Manufacturing Enterprise’s Internet Strategic Transformation Based on Back Propagation Artificial Neural Network

#### Construction of Single Hidden Layer Back Propagation Network Model

This study used the BP artificial neural network with a hidden layer when constructing the causal relationship evaluation model of the risk influencing factors of the manufacturing enterprise’s Internet strategic transformation.

The network topology is composed of p nodes, and these nodes actually represent x1, x2,…, xp in the hidden layer, there are a total of q nodes, and the parameter wij (*j* = 1, 2,…, p; i = 1, 2,…, q) is the connection between the j-th input node and the i-th hidden layer node The weight, θi (i = 1, 2,…, q) represents the offset value of the i-th hidden layer node, αi (i = 1, 2,…, q) actually communicates the hidden layer node and the output layer, and is the connection weight between the two. The hidden layer activation function is constructed based on the logitic S function to obtain the formula:


(1) $$f\,\left(x\right)=\frac{1}{1+\exp\left(-x\right)}$$

Like other functions, the logitic S-type function also has a special operating mechanism. Through the substitution of values, the final result is limited to (0, 1). The number of nodes in the output layer is 1, which is the evaluation value y of the manufacturing enterprise’s Internet strategic transformation risk. Through the output weighted input plus the deviation value, the activation function of the hidden layer and the output layer is constructed, namely:


(2) $$y=f\,\left(\sum_{k=1}^{n}w_{k}\,x_{k}+\theta \right)=\sum_{k=1}^{n}w_{k}\,x_{k}+\theta$$

From the constructed network topology, the function between the output layer and the input layer is determined, that is, the functional relationship between y and (x1, x2,…, xp):


(3) $$y=a_{0}+\sum_{i=1}^{q}a_{i}\,g\,\left(\theta_{i}+\sum_{j=1}^{p}w_{i\,j}\,x_{j}\right)$$

Equation (3) can also be simplified and described as:


(4) $$y=f\,\left(x_{1},x_{2},\ldots ,x_{p},w\right)$$

Equation (4) shows that the relationship between the risk function of the manufacturing enterprise’s Internet strategy transformation contains two variables, namely (x1, x2,…, xp) and the network parameter w. Therefore, it can be seen that this function relationship is affected by the network topology and the neuron The influence of activation function. To change the formula of 3, add random disturbance term:


(5) $$y=f\,\left(x_{1},x_{2},\ldots ,x_{p},w\right)+\varepsilon$$

In the end, the functional relationship of the constructed BP artificial neural network evaluation model is shown to be essentially a multiple nonlinear regression model, which not only avoids the multiple collinear problem of regression analysis, but also combines the characteristics of the research object to improve the accuracy of the model.

#### Construction of Risk Evaluation Model Based on Back Propagation Artificial Neural Network

##### Input Layer

The risks of manufacturing enterprises’ Internet strategic transformation can be comprehensively analyzed and evaluated through a series of observable qualitative or quantitative indicators. On the premise that the risk evaluation index is determined, the number of nodes in the input layer of the neural network is also determined. According to the risk evaluation index system constructed in the first section of this chapter, the observation index items are determined as the input nodes of the network to form the input layer of the network. The value of this layer is 18.

##### Hidden Layer

The network structure studied in this paper contains three layers. To ensure the operability of the network, a reasonable and moderate number of hidden layer nodes must be determined. Generally, if the number of nodes is too small, it is easy to reduce the adaptability and fault tolerance of the network, making the network usability narrower, unable to deal with complex problems, and unable to achieve the desired results; on the contrary, if there are too many nodes, it is easy. When the data do not converge, on the one hand, the network training time is prolonged, and, on the other hand, it is not conducive to the identification of effective models. In contrast, if the number of nodes are too many, the data will not converge. On the one hand, the network training time is prolonged, and, on the other hand, it is not conducive to the identification of effective models. Therefore, it is necessary to combine accuracy and generality to determine the number of nodes in the hidden layer. At present, there is no unified method for determining the number of hidden layer nodes. We can refer to the following empirical formulas summarized by previous scholars to determine the minimum and maximum number of hidden layers to ensure the fastest convergence:


(6) $$N\,u\,m\,b\,e\,r\,o\,f\,h\,i\,d\,d\,e\,n\,l\,a\,y\,e\,r\,n\,o\,d\,e\,s=\sqrt{m+n}+a$$

In the formula, m and n, respectively represent the number of nodes in the input layer and output layer, a represents a constant, and the value range is [1,10].

Based on the above considerations and many experiments, this study determined that the hidden layer of the network finally contains four nodes.

##### Output Layer

Because there is a corresponding relationship between the output node and the evaluation structure, it is necessary to determine the expected output in advance before using the network evaluation model. Combined with empirical data or the expert scoring method, the expected output value can be determined in advance. This study adopted the latter method to determine the expected value and divides the risks of manufacturing enterprises’ Internet strategy transformation into four levels, namely security (1), sub-security (2), risk (3), and high risk (4).

The number of neurons in the output layer is *n* = 4 from the risk division method of the manufacturing enterprise’s Internet strategy transformation. Therefore, we constructed the 18-4-4 BP artificial neural network structure in this study. Application of Risk Evaluation Model for Manufacturing Enterprise’s Internet Strategic Transformation.

### Use of Evaluation Models

As the research on the risks of the Internet strategic transformation of manufacturing companies is still in its infancy and there is no corresponding historical data, this study used the empirical data in the questionnaire as a sample for network training and built a BP artificial neural network evaluation model for the risk of manufacturing companies’ Internet strategic transformation based on SPSS19.0.

Taking 18 questionnaire survey indicators as the input layer and four categories of transformation risk as the output layer, after software automatic optimization and selection, a hidden layer with four nodes was finally determined. To prevent the network from over-training and to ensure the “normal” operation of the neural network, a certain proportion of test samples were set. The sample data were randomly allocated into three parts: test, training, and maintenance. These accounted for 54.9, 14.5, and 30.6%, respectively, as shown in Table 1.

**TABLE 1:** Summary table of case processing of BP artificial neural network evaluation model.

		N	Percentage
Sample	Training	212	54.9%
	Test	56	14.5%
	Maintain	118	30.6%
Effective	386	100.0%	
Excluded	0		
Total	386		

The constructed network information is shown in Table 2.

**TABLE 2:** Summary table of BP artificial neural network evaluation model.

Training	Cross entropy error	9.028
	Percentage error prediction	0.5%
	Discontinued rules	1 consecutive steps with unreduced errors
	Training period	00:00:00.087
Test	Cross entropy error	19.980
	Percentage error prediction	8.9%
Maintain	Percentage error prediction	6.8%

*Dependent variable: Risk.*

The error is reduced after one step for the algorithm; therefore, the estimation algorithm stops and reaches a more ideal state, as shown in Table 3.

**TABLE 3:** Classification and display table of training results of BP artificial neural network evaluation model.

Sample	Observed	Predicted				
		1	2	3	4	Correct percentage
Training	1	0	1	0	0	0.0%
	2	0	11	0	0	100.0%
	3	0	0	141	0	100.0%
	4	0	0	0	59	100.0%
	Percent of total	0.0%	5.7%	66.5%	27.8%	99.5%
Test	1	0	1	0	0	0.0%
	2	0	1	3	0	25.0%
	3	0	0	40	1	97.6%
	4	0	0	0	10	100.0%
	Percent of total	0.0%	3.6%	76.8%	19.6%	91.1%
Maintain	1	0	1	0	0	0.0%
	2	0	6	1	0	85.7%
	3	0	1	75	3	94.9%
	4	0	0	2	29	93.5%
	Percent of total	0.0%	6.8%	66.1%	27.1%	93.2%

*Dependent variable: Risk.*

**FIGURE 3:**
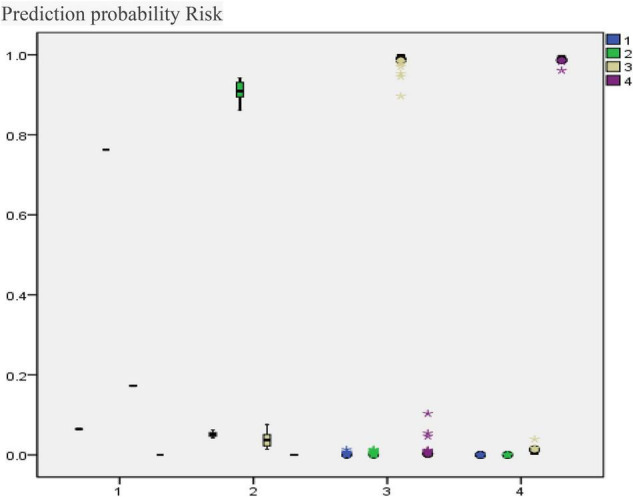
Observation and prediction diagram of BP artificial neural network evaluation model.

It can be seen from Table 3 that the training and testing samples approach or reach 100% accuracy, and the test samples also reach over 90% accuracy. The model fits well, as shown in Figure 3.

#### Prediction Probability Risk

Figure 3 shows that for each category of transition risk, only a few cases have classification errors, indicating that the model has a good predictive effect and can be used for risk assessment and prediction.

### Applications

#### Sample Company Selection

There are many types of manufacturing companies, but there are common key factors for manufacturing companies’ Internet strategic transformation. This study selected a food manufacturing company in Hubei as a sample for empirical analysis.

Xiaogan Seasame Chips & Rice Beverage Co., Ltd., Xiaogan, China, which processes agricultural products, was established in September 2003 from the original food manufacturing plant. The company covers an area of more than 20,000 square meters and has 412 employees, including professional and technical personnel. A total of 102 people, with more than 60 sets of modern food production lines and supporting equipment. The company has passed quality management certifications such as ISO9001, HACCP, and export registration certificates. The products are not only sold in good domestic condition, but also exported to Singapore, the United Kingdom, the United States, and other European regions. The company was awarded the “Advanced Key Leading Enterprise of Agricultural Industrialization in Hubei Province” and has received successive ratings of “Consumer Trustworthy Product of Hubei Province” and “Consumer Satisfaction Unit of Hubei Province.” It was also awarded honorable titles for a typical food manufacturing enterprise, such as “Contract-honoring and Credit Enterprise” and “Civilized and Honest Private Enterprise.”

In recent years, the company has focused on Internet applications and has successively invested more than 80 million Yuan in intelligent transformation of production equipment. Internet technology has not only been applied in many areas such as production, marketing, and after-sales services, but has also been used to jointly develop intelligent robots for production with scientific research institutes. Installation and debugging are in progress. Senior executives of the company say they will increase investment in the future to further transform the company from “manufacturing” to “intelligent manufacturing” and unswervingly follow the path of Internet strategic transformation. Therefore, this is a representative company to select to conduct empirical research on the risks of manufacturing enterprises’ Internet strategic transformation.

#### Data Sources

The data collection of this study was conducted by the author at the food manufacturing company by field investigation, with invited middle and senior managers and relevant experts of the company. Brainstorming methods were used to discuss the main indicators of the Internet strategic transformation risks of the manufacturing company. The scores of various indicators of the enterprise are shown in Table 4.

**TABLE 4:** Evaluation table of managers and experts of a food manufacturing company.

Index	A1	A2	A3	A4	A5	A6	B1	B2	B3	B4	B5	B6	C1	C2	C3	C4	C5	C6
Score	5	3	5	2	2	1	3	3	5	4	3	3	3	3	4	4	4	4

#### Evaluation Results Analysis

By consulting the model established above, the probability of each category of risk in the manufacturing company’s Internet strategy transformation is predicted to be (0.000, 0.000, 0.999, 0.000), and the predicted value is 3, which means it is in a “risk” state.

From field investigations and interviews at the company, we learned that in the process of the company’s intelligent transformation of production equipment, the original main equipment utilization rate was low. The cost of equipment sinking was high, according to the vice president responsible for the transformation of production equipment. According to the manager, in some production line renovations, the length of the original plant was inappropriate, and another plant had to be built, which required a large investment. In the context of the current downward pressure on the economy, it is more difficult to raise financing, and the financing cost is high. Because of a lack of previous experience, much of the equipment could not be directly introduced and needed to be jointly developed with scientific research institutes and equipment manufacturers. It sometimes takes many attempts to succeed, which leads to high costs. Although this company has introduced specialized Internet technology talents, most of the production managers are weak in Internet application skills and lack flexible management abilities; therefore, they cannot give full play to the efficiency and benefits brought about by the corporate Internet strategy transformation. Consequently, during the company’s Internet strategic transformation, equipment flexibility risks, financing management risks, Internet technology risks, and flexible management risks are relatively high, which is in line with the reality of a “risk” state. Judging from the application of the above examples, the risk evaluation model of manufacturing enterprise Internet strategy transformation established in this article is objective and practical.

## Data Availability Statement

The raw data supporting the conclusions of this article will be made available by the authors, without undue reservation.

## Ethics Statement

The studies involving human participants were reviewed and approved by the Research committee of School of economics and management Hubei Engineering University. The patients/participants provided their written informed consent to participate in this study.

## Author Contributions

HH was responsible for conceptual development, write-up, and overall supervision. GZ was responsible for methodology and data section. Both authors contributed to the article and approved the submitted version.

## Conflict of Interest

The authors declare that the research was conducted in the absence of any commercial or financial relationships that could be construed as a potential conflict of interest.

## Publisher’s Note

All claims expressed in this article are solely those of the authors and do not necessarily represent those of their affiliated organizations, or those of the publisher, the editors and the reviewers. Any product that may be evaluated in this article, or claim that may be made by its manufacturer, is not guaranteed or endorsed by the publisher.
